# Effects of UV/H_2_O_2_ Degradation on the Physicochemical and Antibacterial Properties of Fucoidan

**DOI:** 10.3390/md22050209

**Published:** 2024-05-03

**Authors:** Zhicheng He, Biyang Zhu, Lijuan Deng, Lijun You

**Affiliations:** School of Food Science and Engineering, South China University of Technology, Guangzhou 510640, China; ncuspyhezhicheng@163.com (Z.H.); fezhuby@mail.scut.edu.cn (B.Z.); liidlj007@163.com (L.D.)

**Keywords:** fucoidan, antibacterial activity, degradation, minimum inhibitory concentration, minimum bactericidal concentration

## Abstract

The applications of fucoidan in the food industry were limited due to its high molecular weight and low solubility. Moderate degradation was required to depolymerize fucoidan. A few studies have reported that fucoidan has potential antibacterial activity, but its antibacterial mechanism needs further investigation. In this study, the degraded fucoidans were obtained after ultraviolet/hydrogen peroxide treatment (UV/H_2_O_2_) at different times. Their physicochemical properties and antibacterial activities against *Staphylococcus aureus* and *Escherichia coli* were investigated. The results showed that the average molecular weights of degraded fucoidans were significantly decreased (up to 22.04 times). They were mainly composed of fucose, galactose, and some glucuronic acid. Fucoidan degraded for 90 min (DFuc-90) showed the strongest antibacterial activities against *Staphylococcus aureus* and *Escherichia coli*, with inhibition zones of 27.70 + 0.84 mm and 9.25 + 0.61 mm, respectively. The minimum inhibitory concentrations (MIC) were 8 mg/mL and 4 mg/mL, respectively. DFuc-90 could inhibit the bacteria by damaging the cell wall, accumulating intracellular reactive oxygen species, reducing adenosine triphosphate synthesis, and inhibiting bacterial metabolic activity. Therefore, UV/H_2_O_2_ treatment could effectively degrade fucoidan and enhance its antibacterial activity.

## 1. Introduction

Food safety is always the focus of the global society, among which, foodborne pathogens are one of the most important sources of risk to food safety and human health. Some foodborne pathogens, such as *Staphylococcus aureus* (*S. aureus*) and *Escherichia coli* (*E. coil*), could cause food spoilage and lead to the development of diseases. Antibiotics were effective ways to resist pathogenic microorganisms and treat foodborne diseases, but their prophylactic use was limited by drug resistance and side effects [1]. Therefore, there is an urgent need to develop other antibacterial materials from natural resources. Some seaweeds have been reported to have potential antibacterial activities to inhibit bacterial infection in complex and variable aquatic environments. For example, marine sulfated polysaccharides prepared from green macroalgae *Ulva armicicana* had antibacterial activities against Gram-positive and Gram-negative bacteria [2]. A sulfated polysaccharide extracted from *Chlamydomonas phine* showed antibacterial and antibiofilm potential [3].

In recent years, fucoidan (Fuc) extracted from brown algae has been proven to have various biological activities, such as immune regulation [4], anti-inflammatory [5], antioxidant [6], and antibacterial activities [7]. However, its complex structure and high molecular weight (Mw) lead to low solubility, which limits its practical applications [8]. The structure–activity relationship of polysaccharides has shown that the low molecular weight of fucoidan might have better biological activities, such as anti-inflammatory and anti-cancer activities [9]. Therefore, it is necessary to degrade fucoidan to increase its solubility and improve its biological activities.

Our research group previously found that ultraviolet/hydrogen peroxide (UV/H_2_O_2_) treatment could be used to degrade marine polysaccharides. For example, it could effectively reduce the molecular weight of *Sargassum fusiforme* polysaccharide and enhance its anti-inflammatory and anti-photoaging activities [10,11]. Whether the UV/H_2_O_2_ treatment could degrade fucoidan and improve its activity needs to be explored. Therefore, in this study, fucoidan was treated by UV/H_2_O_2_ degradation. The structural characteristics of Fuc and degraded fucoidan (DFucs) were studied, and their antibacterial activities against *Staphylococcus aureus* ATCC 25923 and *Escherichia coli* ATCC 25922 were also evaluated. The results will lay a foundation for exploring fucoidan as a natural ingredient with good antibacterial activity.

## 2. Results and Discussion

### 2.1. pH Values and Molecular Weights

**Figure 1:**
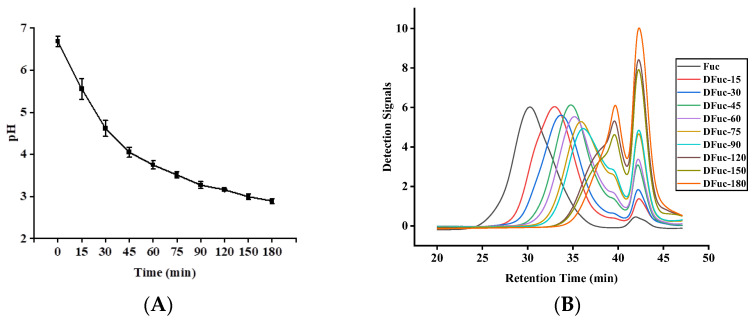
The pH values (**A**) and average molecular weights (**B**) of the degraded fucoidans.

The pH values of fucoidan degraded by UV/H_2_O_2_ after different treatment times are shown in Figure 1A. As the treatment time prolonged, the pH values continuously decreased. It decreased from 6.69 ± 0.12 to 2.89 ± 0.06 after degradation for 180 min. The decrease in pH values was mainly attributed to the electron-withdrawing of free radicals to produce H^+^, as well as the production of acidic substances in fucoidan solutions. Therefore, the change in pH values could reflect the degradation effect of fucoidan [12]. In this study, the pH values decreased rapidly from 6.69 ± 0.12 to 3.16 ± 0.02 at 0–120 min, indicating the severe degradation of fucoidan. At 120–180 min, the pH values tended to stabilize, indicating that the content of hydroxyl radicals (·OH) decreased and most of the main sites attacked by free radicals in polysaccharides had been broken after degradation for 120 min [13]. The above results were consistent with those of the study by Yang et al. [14].

As shown in Figure 1B, the molecular weights of the fucoidan degraded by UV/H_2_O_2_ decreased with treatment time. The curves showed that the molecular weights decreased rapidly at first and then more slowly. After 120 min, the molecular weights of fucoidan decreased from 530.7 kDa to 3.6 kDa. Within 120–180 min, the molecular weights of the fucoidan had decreased to 3.6–3.4 kDa (Table 1). The decrease in molecular weights of fucoidan was attributed to the disruption of glycosidic bonds by ·OH free radicals generated from H_2_O_2_ under UV irradiation [15,16].

It has shown that the molecular weight of fucoidan had a significant impact on their biological activities. Jun et al. found that the antibacterial activity of fucoidan against dental plaque bacteria was enhanced after heat treatment (121 °C, 15 min) and molecular weights of fucoidan reduced from 74.2 kDa to 13.9 kDa [17]. Liu et al. reported that fucoidan fragments with molecular weights less than 6 kDa had better antibacterial activities against *E. coli* and *S. aureus*, compared to the fragments with molecular weights more than 6 kDa [18]. Rutz et al. reported that fucoidan after degradation by H_2_O_2_-Vc treatment had better antibacterial activity against *E. coli* and *S. aureus* compared with the original fucoidan [19]. Based on the previous studies, we speculated that fucoidan with lower molecular weight might have better antibacterial activity.

### 2.2. Chemical Composition

**Figure 2:**
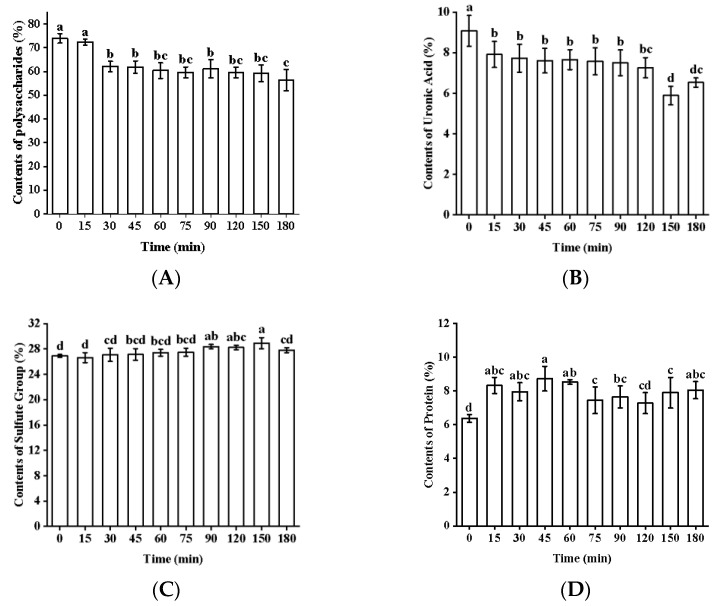
Chemical compositions of fucoidans. The contents of total sugar (**A**), uronic acid (**B**), protein (**C**), and sulfate group (**D**). Different lowercase letters mean significantly different (*p* < 0.05).

The chemical compositions of the degraded fucoidans are shown in Table 2 and Figure 2. With the prolongation of treatment time, the total sugar and uronic acid contents of fucoidans decreased by 23.7% and 28.0%, respectively, while the sulfate group contents of sugars increased by 6.6%. The protein content did not change significantly. The OH free radical attacked the H atom of the polysaccharide C bond, forming a carbon center free radical of the polyethylene group. The free radicals attacked polysaccharides, ultimately leading to glycosidic bond cleavage and chemical composition changes [20,21]. The decrease in the content of uronic acid was due to the decarboxylation of polysaccharides under intense degradation conditions [22]. Connection sites between sulfate groups and polysaccharides were less susceptible to free radical attacks, but the decrease in total sugar content led to a relative increase in the contents of sulfate groups [13]. The sulfate group contents of polysaccharides were a key factor in their biological activities [23]. Liu et al. also found that fucoidan with higher sulfate group contents showed stronger antibacterial activity [18]. Therefore, the increase in the sulfate group contents of the degraded fucoidans might have contributed to their enhanced antibacterial activity.

### 2.3. Monosaccharide Composition

**Figure 3:**
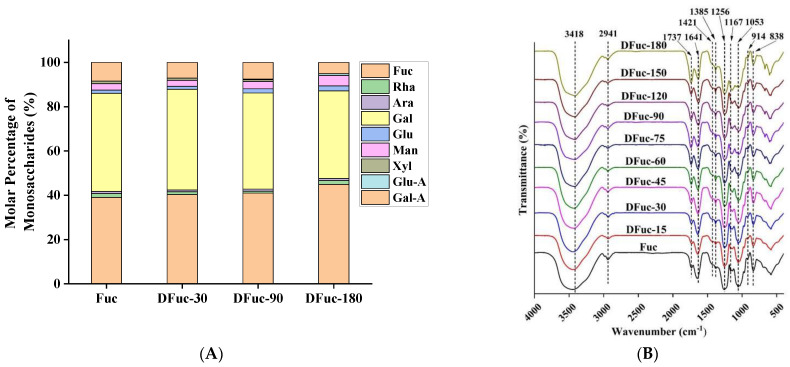
Monosaccharide composition (**A**) and infrared spectra (**B**) of degraded fucoidans.

The monosaccharide compositions of degraded fucoidans are shown in Table 3 and Figure 3A. The results showed that the main monosaccharide components of fucoidans were galactose and fucose, accounting for 83.5–84.4%, followed by glucuronic acid and mannose. Fuc and DFucs also contained small amounts of rhamnose, arabinose, glucose, and xylose, which were consistent with the main components of fucoidan determined by Saravana et al. [24]. The main monosaccharide composition types of DFucs were the same as Fuc, indicating that treatment of UV/H_2_O_2_ did not change the main monosaccharide types of polysaccharides. As the processing time increased, the contents of fucose and mannose increased and the contents of galactose and glucuronic acid decreased, indicating that galactose and glucuronic acid were the main attacking sites by free radicals in the UV/H_2_O_2_ system [18]. However, the changes in other monosaccharides did not show any rules, which might be related to the complex structure of fucoidan and the randomness of attacking sites by free radicals.

### 2.4. Fourier Transform Infrared Spectroscopy

Infrared absorption spectroscopy could be used to quantitatively analyze some functional groups of polysaccharides, such as O-H, N-H, and C-O [25]. As shown in Figure 3B, the absorption peaks at 3418 and 2941 cm^−1^ were caused by O-H stretching vibration and -CH_3_ stretching vibration, respectively, both of which were the characteristic absorption peaks of polysaccharides [26]. The peaks at 1737 cm^−1^ and 1385 cm^−1^ were caused by the stretching vibration of the C=O double bond and the stretching vibration of C-H, respectively. The combination of the C=O and C-O bond, proved by 1639 cm^−1^ and 1421 cm^−1^, indicates the presence of uronic acid [27]. In addition, absorption at 1256 cm^−1^ (S=O stretching vibration) and 837 cm^−1^ corresponded to the stretching vibration of the S=O and C-O-S bonds, respectively, indicating the presence of sulfate groups [28]. The strong absorption peak at 1053 cm^−1^ was generated by the stretching vibration of the C-O-H bond, which was a characteristic peak of the pyran ring, while the weak absorption peak at 914 cm^−1^ was the characteristic peak of the *β*-glycosidic bond [29]. The Fourier transform infrared spectroscopy analysis results showed that Fuc and DFucs were typical sulfated polysaccharides, containing pyran rings and *β*-glycoside bonds. Keisham et al. reported that the pyran ring was a universal structural unit that constituted a variety of biologically active natural substances, including antibacterial activity [30]; therefore, the antibacterial activity of fucoidan could be investigated in the following study.

### 2.5. Antibacterial Zones

**Figure 4:**
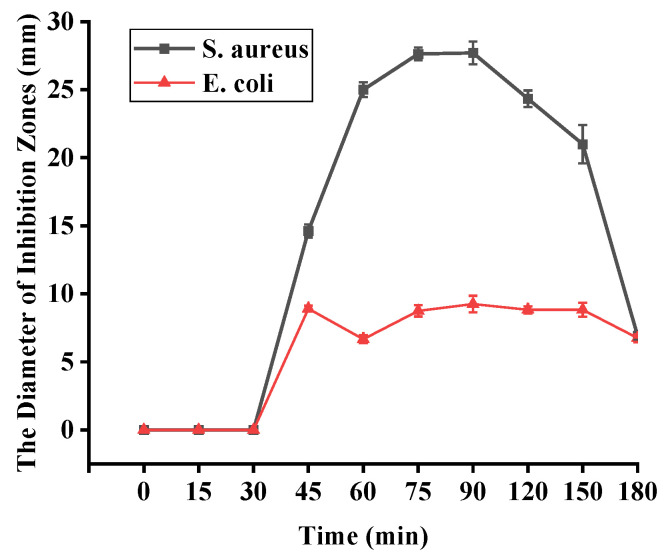
The diameter of inhibitory zones against *S. aureus* and *E. coli* treated with degraded fucoidans.

The antibacterial activities of degraded fucoidans were studied. As shown in Figure 4, DFuc-90 showed the maximum inhibitory zones against *S. aureus* and *E. coli*, which were 27.70 ± 0.84 mm and 9.25 ± 0.61 mm, respectively (Supplementary Figure S1A,B). The antibacterial activity of degraded fucoidan increased up to 90 min but then decreased with further degradation. This might be because the molecular weight of fucoidan was decreased and -CO, -OH, -COO, and -CH were exposed, which had good chelating ability towards metal ions, thereby affecting or inhibiting bacterial absorption of nutrients and metal ions [31]. After 90 min, the antibacterial activity of degraded fucoidan decreased. It was speculated that the spatial conformations of fucoidan were broken after a long time of degradation, which might decrease its antibacterial activity. This indicated that fucoidan might require a certain degree of polymerization and molecular weight to show antibacterial activity. Meanwhile, Liu et al. concluded that fucoidan with low molecular weight (5–50 kDa) and high sulfate group content (over 20%) had stronger biological activity [18]. Similarly, the degraded fucoidan with molecular weight 20–35 kDa and sulfate group content (over 27%) showed good antibacterial activities in our study. These DFucs expressed good antibacterial activities against both *S. aureus* and *E. coli*. Among the samples, DFuc-90 showed the strongest antibacterial activity.

### 2.6. Analysis of Antibacterial Activity of DFuc-90

#### 2.6.1. MIC and MBC

**Figure 5:**
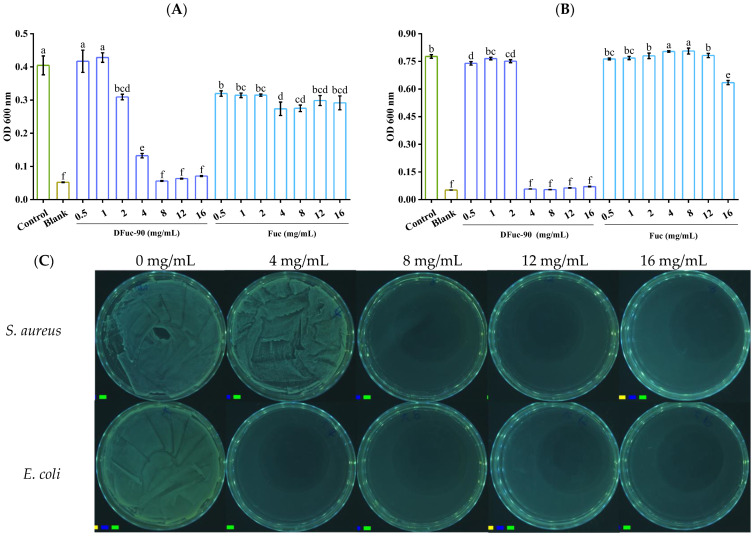
The MIC of DFuc-90 against *S. aureus* (**A**) and *E. coli* (**B**), and the MBC of DFuc-90 against *S. aureus* and *E. coli* (**C**). Different lowercase letters mean significantly different (*p* < 0.05).

Inhibitory zone and minimum inhibitory concentration (MIC) have been used to study the inhibitory antibacterial activity of polysaccharides, but methodological differences hinder the reliable estimation of inhibitory polysaccharide analysis comparison [32]. Therefore, the MICs of DFuc-90 against *S. aureus* and *E. coli* were determined. As shown in Figure 5A,B, the MICs of DFuc-90 against *S. aureus* and *E. coli* were 8 mg/mL and 4 mg/mL, respectively. The minimum bactericidal concentrations (MBCs) were 8 mg/mL and 4 mg/mL, respectively (Figure 5C). In addition, Liu et al. found that the MICs of the degraded *Laminaria japonica* fucoidan against *S. aureus* and *E. coli* were 12.5 mg/mL and 8 mg/mL, respectively, which were degraded through high-temperature and high-pressure hydrolysis (121 °C, 0.103 MPa, 40 min) [18]. Ashayerizadeh et al. also prepared low molecular weight fucoidan from *Sargassum tenerrimum* using the same method, and its MIC against *S. aureus* and *E. coli* were 12.5 mg/mL and 12.5 mg/mL, respectively [7]. In our work, the MICs of degraded fucoidan obtained by UV/H_2_O_2_ treatment against bacteria were 8 mg/mL and 4 mg/mL, respectively, which were all lower than the MIC of fucoidan mentioned above. It indicated that UV/H_2_O_2_ degradation had more advantages in preparing antibacterial polysaccharides, compared to traditional high-temperature and high-pressure hydrolysis degradation.

#### 2.6.2. Bacterial Growth Curve

**Figure 6:**
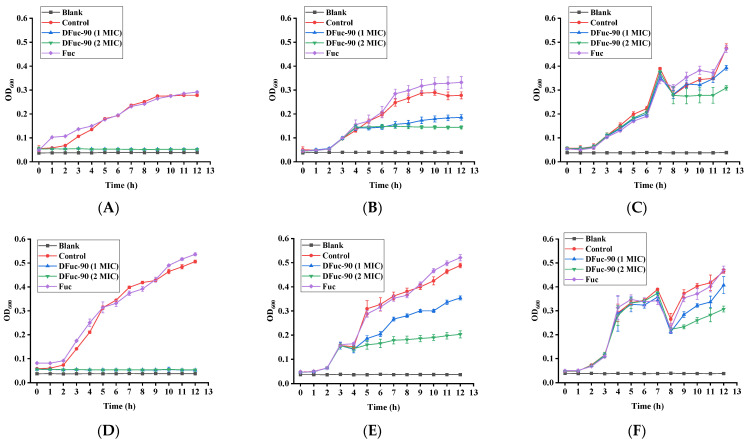
Effects of Fuc and DFuc-90 on the growth curves of *S. aureus* at 0 h (**A**), 4 h (**B**), and 8 h (**C**), respectively. Effects of Fuc and DFuc-90 on the growth curves of *E. coli* at 0 h (**D**), 4 h (**E**), and 8 h (**F**), respectively.

DFuc-90 significantly inhibited the growth of *S. aureus* and *E. coli*, and 2 × MIC of DFuc-90 had a better inhibitory effect on bacteria than 1 × MIC. As shown in Figure 6, after adding 2 × MIC and 1 × MIC of DFuc-90 at 0 h, respectively, the bacteria lost their vitality and the OD_600_ values no longer increased. However, the OD_600_ values of the Fuc group were similar to that of the control group, indicating that Fuc did not affect the bacteria. After adding 2 × MIC and 1 × MIC of DFuc-90 at 4 and 8 h, the growth of *S. aureus* and *E. coli* was inhibited. The OD_600_ values in Figure 6C and F at 8 h were measured after the addition of DFuc-90. After mixing the bacterial suspension in the well plate with a sterile clear sample solution, the bacterial density and the absorbance values of the mixtures decreased. The bacterial exponential growth rate had decreased significantly, which was consistent with the previous results [33,34]. Compared to adding DFuc-90 at 4 h, the antibacterial ability of DFuc-90 was lower than after adding DFuc-90 at 8 h. It was likely that bacteria had been in the logarithmic phase at 4 h, which required a large amount of nutrients to meet their exponential division, while they were more sensitive to external stimuli. It was speculated that the logarithmic phase of bacteria was the main stage for DFuc-90 to show antibacterial activity. Zhang et al. also found that *Cordyceps cicadae* polysaccharide showed the strongest antibacterial activity when *E. coli* grew in the logarithmic phase [35].

#### 2.6.3. Bacterial Cell Viability and Metabolic Activity

**Figure 7:**
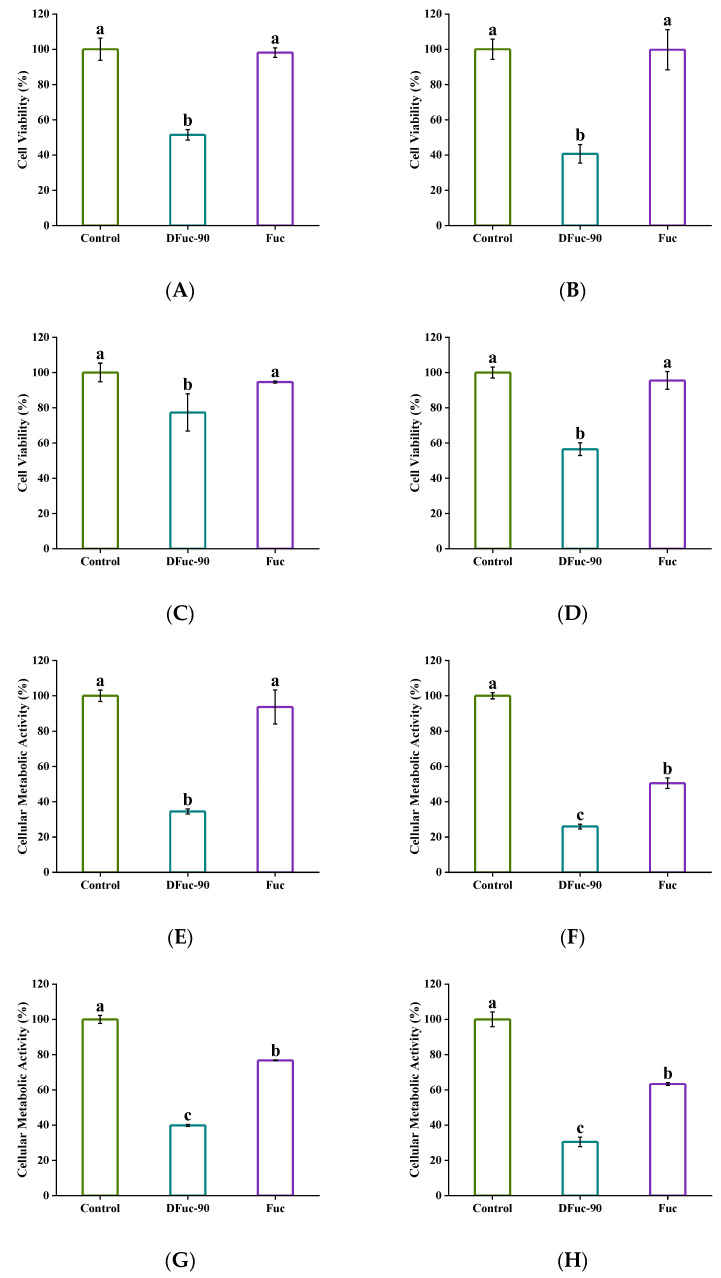
Effect of 2 × MIC of DFuc-90 on the bacterial cell viabilities and metabolic activities after incubation for 3 and 24 h, respectively. (**A**) Cell viability of *S. aureus* after 3 h; (**B**) cell viability of *S. aureus* after 24 h; (**C**) cell viability of *E. coli* after 3 h; (**D**) cell viability of *E. coli* after 24 h; (**E**) metabolic activity of *S. aureus* after 3 h; (**F**) metabolic activity of *S. aureus* after 24 h; (**G**) metabolic activity of *E. coli* after 3 h; (**H**) metabolic activity of *E. coli* after 24 h. Different lowercase letters mean significantly different (*p* < 0.05).

The 3-[4,5-dimethylthiazol-2-yl]-2,5- diphenyltetrazolium bromide (MTT) assay was normally used to evaluate the number of live bacteria. As shown in Figure 7A–D, there was no significant difference in the survival rate of bacteria between the Fuc group and the control group (*p* > 0.05). However, after incubation with 2 × MIC of DFuc-90 for 3 and 24 h, the survival rates of *S. aureus* decreased by 48.52% and 59.40%, respectively. The survival rates of *E. coli* decreased by 22.70% and 43.50%, respectively. The results were consistent with the study by Zhang et al., which showed that the survival rate of *E. coli* was reduced by 29.20% after incubation with *Armillariella tabescent* polysaccharide [36].

Live bacteria produce an activated hydrogen ion under the action of dehydrogenase, which can reduce the iodonitrotetrazolium chloride (INT) to stable formazan and the rate of formazan formation can be used to predict the metabolic activity of bacteria [37]. After incubation with 2 × MIC of DFuc-90 for 3 and 24 h, the metabolic activities of *S. aureus* decreased by 65.60% and 74.04%, respectively, compared to the control group, while the metabolic activities *of E. coli* decreased by 60.11% and 69.5%, respectively (Figure 7E–H). Previous studies showed that antibacterial polysaccharides could affect the metabolic activity of bacteria [38]. The overall bacterial metabolic activities after 24 h were lower than that after 3 h of cultivation, which may be due to the bacteria having entered the aging stage and having lower cellular metabolic activities.

#### 2.6.4. Bacterial Cell Wall Integrity

**Figure 8:**
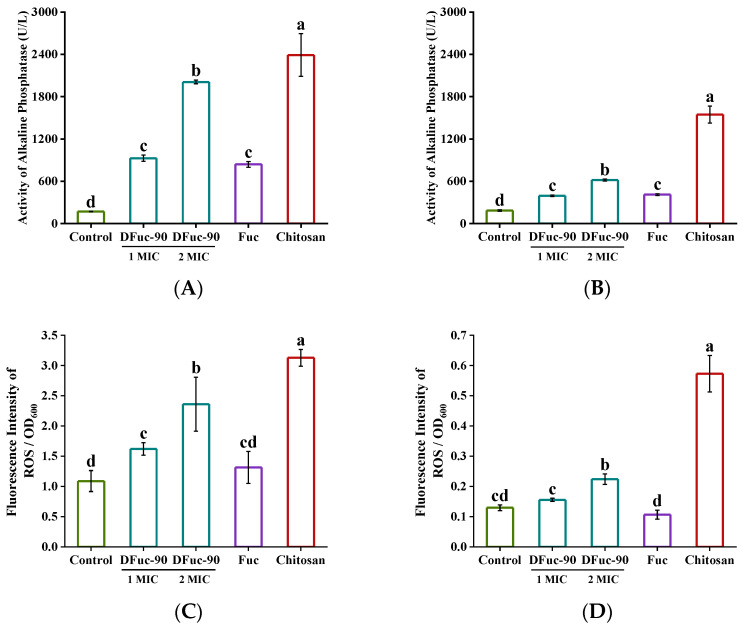

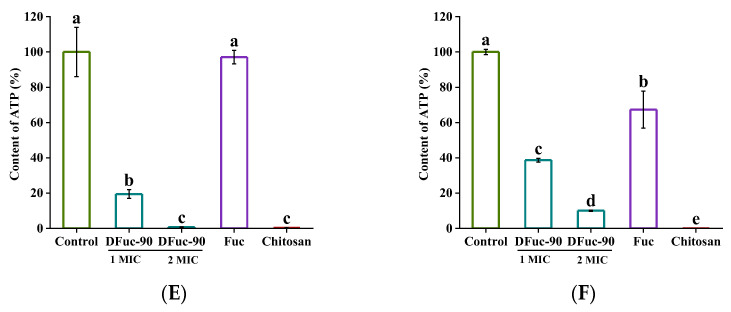
Effect of DFuc-90 on the bacterial AKP activity, ROS, and ATP. (**A**) AKP activity of *S. aureus*; (**B**) AKP activity of *E. coli*; (**C**) ROS content of *S. aureus*; (**D**) ROS content of *E. coli*; (**E**) ATP content of *S. aureus* after 3 h; (**F**) ATP content of E. coli. Different lowercase letters indicated significant differences (*p* < 0.05).

Alkaline phosphatase (AKP) is an enzyme located between the cell membrane and the cell wall of the bacteria. It would leak out when the integrity of the bacterial cell wall was disrupted [39]. As shown in Figure 8A,B, the AKP activity of *S. aureus* bacterial extracellular fluid increased by 4.42 times and 10.81 times after 5 h of incubation with 1 × MIC and 2 × MIC of DFuc-90, respectively. The same phenomenon existed in *E. coli* with an increase of 1.14 times and 2.24 times, respectively, indicating that DFuc-90 destroyed the cell walls of *S. aureus* and *E. coli*. After treating *S. aureus* and *E. coli* with Fuc for 5 h, the AKP activity increased by 3.80 times and 1.28 times, respectively, which might be due to the osmotic pressure effect of high concentrations of polysaccharides. However, the AKP activity of the Fuc group supernatant was lower than that of the DFuc-90 group at the same concentration. It was speculated that low molecular weight fucoidan obtained by UV/H_2_O_2_ degradation was more likely to interact with bacterial cell walls to damage bacteria and inhibit bacterial growth. Zhang et al. found that cordyceps polysaccharide increased the AKP activity of *E. coli* and inhibited its growth [35]. Another scholar Zhang also found that mycelium polysaccharide could increase extracellular AKP activity and inhibit the growth of *S. aureus* and *E. coli*. [36]. Therefore, the polysaccharides could show antibacterial activity by disrupting bacterial cell walls.

#### 2.6.5. Bacterial Reactive Oxygen Species

Reactive oxygen species (ROS) was a class of oxygen-containing active molecules, including hydrogen peroxide, superoxide, pure oxygen, and hydroxyl radicals. Abnormal levels of ROS led to lipid oxidation, protein oxidation, DNA strand breakage, and base modification errors, which were generally recognized as the cause of significant cell damage [40]. As shown in Figure 8C,D, there was a significant difference in ROS contents between the 2 × MIC DFuc-90 treatment group and the control group (*p* < 0.05), indicating that the treatment of DFuc-90 would lead to the accumulation of intracellular ROS in bacteria. The accumulation of ROS could cause changes in the redox balance in cells and lead to a series of cellular dysfunctions, which could explain the antibacterial activity of DFuc-90. The same phenomenon existed in the study by Zhang et al., which showed that mycelium polysaccharide increased the intracellular ROS content of *E. coli* [36].

#### 2.6.6. Bacterial Intracellular ATP

As an important multifunctional nucleotide, intracellular adenosine triphosphate (ATP) is involved in many cellular metabolisms and signal transduction processes. The ATP contents could reflect whether the physiological metabolism process of bacteria is normal. As shown in Figure 8E,F, after bacteria were incubated with 1 × MIC and 2 × MIC of DFuc-90 for 5 h, ATP contents of *S. aureus* decreased by 80.51% and 99.24%, respectively. ATP content of *E. coli* decreased by 61.36% and 81.96%, respectively. The results indicated that DFuc-90 interfered with the normal physiological activities of bacteria by reducing ATP synthesis. The higher the polysaccharide concentration, the better the antibacterial activity.

This study showed that the DFuc-90 destroyed the cell walls of *S. aureus* and *E. coli* and might bind to positively charged membrane proteins due to its polyanion properties. Then the fluidity of the bacterial cell membrane increased, and electrolytes leaked, which led to the depolarization of the bacterial membrane [18,39]. Membrane potential could regulate bacterial membrane transport, adenosine triphosphate synthesis, dynamic communication, and antibiotic introduction [41]. Wang et al. found that the polysaccharide produced by *Chaetomium globosum* CGMCC 6882 could dissipate the membrane potential of *S. aureus*, lend to depolarization of the cell membrane, induce cell apoptosis and programmed cell death [42]. Zhao et al. found that laurel extract induced changes in the plasma membrane potential of *E. coli* O157: H7 and inhibited bacterial growth [43]. He also found that the bacterial membrane potential was dynamic and could hyperpolarize and depolarize. Its dissipation might increase membrane permeability, deplete intracellular ATP, and ultimately lead to bacterial death [43]. In our study, we also observed a decrease in intracellular ATP content in *S. aureus* and *E. coli* after treatment of DFuc-90. At the same time, the increase in intracellular ROS in bacteria indicated that oxidative stress was also involved in the antibacterial activity of DFuc-90 against *S. aureus* and *E. coli*.

## 3. Materials and Methods

### 3.1. Chemicals and Regents

The *Undaria pinnatifida* was collected from Qingdao, Shandong, China. The Mingyue fucoidan (Fuc) was obtained from Qingdao Mingyue Seaweed Group Co., Ltd. (Qingdao, China). Dextran standards (4.66, 12.60, 63.30, 126.00, and 556.00 kDa) and monosaccharide standards (fucose (Fuc), rhamnose (Rha), arabinose (Ara), galactose (Gal), glucose (Glu), mannose (Man), xylose (Xyl), galacturonic acid (Gal-A), glucuronic acid (Glu-A)) were purchased from Sigma-Aldrich Chemical Co. (St. Louis, MO, USA). Phosphate-buffered saline (PBS, pH 7.4) was purchased from Gibco Life Technologies (Grand Island, NY, USA). Luria Bertani (LB) medium was purchased from Guangdong Huankai Microbial Technology Co., Ltd. (Guangzhou, China). AKP assay kit and ROS assay kit were purchased from Beyotime Biotechnology Co., Ltd. (Zhenjiang, China). MTT cell proliferation and cytotoxicity assay kit was obtained from Nanjing Jiancheng Bioengineering Institute (Nanjing, China) and iodonitrotetrazolium chloride (INT) was obtained from Shanghai Macklin Biochemical Technology Co., Ltd. (Shanghai, China). Bac titer-glo ™ microbial cell viability assay kit was purchased from Promega Biotechnology Co., Ltd. (Madison, WI, USA). All reagents used were of analytical grade.

### 3.2. Extract and Degradation of Fucoidan

Fucoidan was obtained from Qingdao Mingyue Seaweed Group Co., Ltd. It was extracted from *Undaria pinnatifida*. The dry *Undaria pinnatifida* powder was extracted in hot water at 100 °C for 4 h with a solid–liquid ratio of 1:50 (*w*/*v*). The residue was removed using filtration and centrifugation and the extraction solution was concentrated using a vacuum rotary evaporator (Hei-VAP values Digital, Heidoph, Nuremberg, Germany). The polysaccharide was precipitated using ethanol. The mixtures were kept at 4 °C for 12 h and then the polysaccharide precipitate was obtained using centrifugation at 8000 rpm for 20 min and dried at room temperature. The fucoidan obtained after the precipitated solution was freeze-dried (vacuum freeze dryer, ALPHA 1–2 LDplus, Christ, Osterode, Germany).

The fucoidan was degraded by UV/H_2_O_2_. Firstly, 25 mL 2.4 mg/mL Fuc solution, 3 mL H_2_O_2_, and 2 mL deionized water were transferred into a 90 mm culture dish. The final concentration of H_2_O_2_ was 100 mmol/L, the irradiation dose was 6500 mJ/cm^2^ and the final concentration of the Fuc was 2 mg/mL. Mixtures were treated in a UV irradiation system (HOPE-MED 8140, Hepu, Beijing, China) for 0, 15, 30, 45, 60, 75, 90, 120, 150, and 180 min, respectively. In the end, catalase was used to remove residual H_2_O_2_ from each group. Finally, the degraded fucoidan obtained after the solution was concentrated using a vacuum rotary evaporator (55 °C, 100 rpm) and then freeze-dried.

### 3.3. pH Values and Molecular Weights

The pH values of Fuc and DFucs solution were measured by pH meter (Five Easy FE20, Metter Toledo, OH, USA). The molecular weights of Fuc and DFucs were determined by high-performance gel permeation chromatography (HPGPC, LC-20 A, Shimadzu Corporation, Tokyo, Japan), which was conducted according to our previous report with some modifications [44].

Shimadzu RID-10A differential detector was used. TSKgel G-6000 PWXL (7.8 × 300 mm), TSKgel G-3000 PWXL (7.8 × 300 mm), and TSKgel protective column (6.0 × 40 mm) were used in series. The mobile phase was 0.02 mol/L KH_2_PO_4_ buffer solution. The column temperature was 40 ± 1 °C and the flow rate was 0.5 mL/min. The injection volume was 30 μL. The polysaccharide samples were dissolved in 0.02 mol/L KH_2_PO_4_ solution and prepared at a concentration of 2 mg/mL. Samples were injected for analysis after passing 0.22 μm filter membranes. The dextrans were used as molecular weight standards, and the molecular weights of Fuc and DFucs were calculated based on the dextran standard curve.

### 3.4. Chemical Compositions

The chemical compositions of degraded fucoidans were determined according to the method of Chang et al. [45]. The contents of total sugar and uronic acid of Fuc and DFucs were determined using the phenol sulphuric acid method (using 1:1 mixture of fucose and galactose as the standard) and m-hydroxyphenyl method (using glucuronic acid as the standard), respectively. The content of sulfate group was determined by the barium sulfate turbidity method (using potassium sulfate as the standard) and the content of protein was determined by the Coomassie brilliant blue colorimetric method (using BSA as the standard).

### 3.5. Monosaccharide Composition

The monosaccharide compositions of Fuc and DFucs were determined by ion chromatography (ICS, 3000, Dionex, Sunnyvale, CA, USA) [46]. Fucoidan samples (10 mg) were mixed with 10 mL trifluoroacetic acid (TFA, 2 mol/L) and hydrolyzed at 105 °C for 6 h. TFA was removed from the hydrolysate by vacuum rotary evaporator (Hei-VAP values Digital, Heidoph, Nuremberg, Germany) with methanol solution and continued rotary drying. The above steps were repeated until there was no acidity. The samples were diluted with 25 mL ultrapure water to a concentration of 0.1–0.5 mg/mL. The solution passed through a 0.22 μm aqueous phase filter membrane to obtain polysaccharide hydrolysis products.

The separation column was CarboPacTM PA20 (3 × 150 mm). The washing solutions contained A phase (ultrapure water), B phase (200 mmol/L NaOH), C phase (500 mmol/L NaAc), and D phase (20 mmol/L NaOH). The flow rate was 0.5 mL/min, and the column temperature was 30 °C. Firstly, the system was washed with 100% B phase for 2 min, then switched to 90% A phase mixed with 10% D phase for 18 min. After sample injection, the system was washed with 90% A phase mixed with 10% D phase for 15 min, then switched to washing containing 70% A phase, 20% C phase, and 10% D phase for 15 min.

### 3.6. Fourier Transform Infrared Spectroscopy

The functional group types of Fuc and DFucs were determined by Fourier transform infrared spectroscopy (FT-IR, Tensor 27, Bruker, Karlsruhe, Germany) [47]. In a word, 1 mg of fucoidan sample was mixed with an appropriate amount of chromatography grade KBr (polysaccharide: KBr ≈ 1:100). Mixtures were pressed to thin slices and then scanned by FT-IR with the scanning wavelength range of 400–4000 cm^−1^.

### 3.7. Bacterial Strains and Culture

Gram-positive bacteria *Staphylococcus aureus* ATCC 25923 and Gram-negative bacteria *Escherichia coli* ATCC 25922 were purchased from the American Type Culture Collection (Manassas, VA, USA). These strains were stored in broth medium at 4 °C and were cultured at 37 °C in LB medium.

### 3.8. Antibacterial Zones

The diameters of the antibacterial zones of Fuc and DFucs against *S. aureus* and *E. coli* were determined by the agar diffusion method [48]. Firstly, 100 μL bacterial suspension (10^6^ CFU/mL) was aspirated onto the surface of LB agar plate and spread evenly, and then sterile blank filter papers were placed (diameter of 6 mm and thickness of 1 mm) on the plate. Then, 20 μL 20 mg/mL degraded fucoidan solutions were added on filter papers, sealed plates were inverted in a biological incubator at 37 °C for 24 h. A plate containing bacterial suspension without Fuc was used as the control and 2% H_2_O_2_ was used as the positive control. The zones of inhibition were measured using a vernier caliper (MITUTOYO, Kawasaki, Japan) horizontally and vertically, taking the average of the two as the diameter of each zoon. Each fucoidan sample was conducted in three parallel experiments, taking the average of the three as the diameter of the antibacterial zone produced by each fucoidan sample.

### 3.9. Antibacterial Activity of DFuc-90

#### 3.9.1. Bacterial MIC and MBC

The MIC and MBC of DFuc-90 against *S. aureus* and *E. coli* were determined by the gradient dilution method [49] and the solid plate culture method [50], respectively. Firstly, 100 μL fucoidan solution and 100 μL bacterial suspension (10^6^ CFU/mL) were mixed and added to 96-well plate. Concentrations of Fuc and DFuc-90 were 16, 12, 8, 4, 2, 1, and 0.5 mg/mL, respectively. The solutions were incubated in an incubator (LRH-150, Yiheng, Shanghai, China) at 37 °C for 24 h. OD_600_ values were measured by a multifunctional enzyme-linked immunosorbent assay by using a reader (ELISA, Filter Max F5, Molecular Devices, San Jose, CA, USA). MIC was the minimum polysaccharide concentration at which there was no bacterial growth after 24 h of cultivation. Then, 100 μL solution without bacterial growth after 24 h was coated and inverted overnight at 37 °C. The minimum concentration corresponding to the blank bacterial colony plate was the MBC of DFuc-90 against bacteria. And 2% H_2_O_2_ and LB medium were used as the positive control and negative control, respectively.

#### 3.9.2. Bacterial Growth Curve

The effect of DFuc-90 on bacterial growth curves was determined using a 96-well plate culture. Firstly, 100 μL bacterial suspensions (10^6^ CFU/mL) were added to the 96-well plate, and then 100 μL polysaccharide solutions were added at 0, 4, and 8 h, respectively, with final concentrations of 1 × MIC and 2 × MIC in the DFuc-90 system. All 96-well plates were incubated at 37 °C in a water bath shaker (THZ-82, Guohua, Changzhou, China) at 180 rpm for 12 h. The OD_600_ values were measured every one hour. The growth curves of *S. aureus* and *E. coli* under different conditions were drawn based on the obtained data, and the antibacterial activities of DFuc-90 were evaluated.

#### 3.9.3. Bacterial Cell Viability and Metabolic Activity

The effects of DFuc-90 on bacterial cell viability were determined by using MTT assay with a commercial kit [51]. Firstly, 500 μL fucoidan solution was mixed with 500 μL bacterial suspension (10^6^ CFU/mL) into a shaking tube with a final concentration of 2 × MIC. Shaking tubes were incubated at 37 °C on a biological shaker (180 rpm) for 24 h. Then, 0.4 mL samples incubated for 3 h and 24 h were centrifuged (4 °C, 8000 rpm, and 5 min), respectively. The bacteria were collected and washed three times with PBS and resuspended in 100 μL PBS and their OD_600_ values were measured. Then, 100 μL bacterial solution and 50 μL 1 × MTT solutions were added to tubes, which were cultured at 37 °C for 4 h. The bacteria were collected and washed three times with PBS. Finally, 0.15 mL dimethyl sulfoxide was added to each tube and shaken on a microplate oscillator for 15 min to completely dissolve the formaldehyde. The OD_570_ values were detected and the effect of DFuc-90 on bacterial cell viability was calculated according to Formula (1).

The metabolic activity was determined by the iodonitrotetrazolium chloride (INT) method [52]. Firstly, 150 μL INT solution dissolved in ultrapure water and methanol solution (1:1) was added to 1350 μL bacterial suspension (10^6^ CFU/mL) with the final concentration of 1 mmol/L. Mixtures were incubated at 37 °C in the dark for 30 min. Then, the bacteria were collected by centrifuging at 4 °C and 10,000 rpm for 5 min. The OD_630_ values were detected and the effect of DFuc-90 on bacterial metabolic activity was calculated according to Formula (2).

(1) $$\mathrm{Bacterial}\;\mathrm{cell}\;\mathrm{viability}\;\mathrm{rate}\;(\%)=\frac{{OD}_{570}/{OD}_{600}(Fuc/DFuc-90)}{{OD}_{570}/{OD}_{600}(Control)}\times 100\%$$

(2) $$\mathrm{Percentage}\;\mathrm{of}\;\mathrm{bacterial}\;\mathrm{cellular}\;\mathrm{metabolic}\;\mathrm{activity}\;(\%)=\frac{{OD}_{630}/{OD}_{600}(Fuc/DFuc-90)}{{OD}_{630}/{OD}_{600}(Control)}\times 100\%$$

#### 3.9.4. Bacterial Cell Wall Integrity

The effect of DFuc-90 on bacterial cell wall integrity was determined by using the AKP assay kit [53]. The bacteria were incubated in LB medium mixed with polysaccharides for 5 h at 37 °C. The final concentrations of Fuc, chitosan systems were 2 × MIC and 1 × MIC, respectively. The DFuc-90 systems were 2 × MIC and 1 × MIC. LB medium was used as the blank control. After centrifuging at 4 °C and 10,000 rpm for 5 min, the AKP activity in the supernatant was determined. In a word, 50 μL supernatant and 50 μL working solutions were added to 96-well plates and then incubated at 37 °C for 10 min. Then 100 μL reaction termination liquid was added to each well. The OD_405_ values were measured and the effect of DFuc-90 on the bacterial cell wall was investigated according to Formula (3).

(3) $$\mathrm{Activity}\;\mathrm{of}\;\mathrm{AKP}=\frac{{OD}_{405}/{OD}_{600}(Fuc/DFuc-90/Chitosan)}{{OD}_{405}/{OD}_{600}(Control)}$$

#### 3.9.5. Bacterial Intracellular Reactive Oxygen Species

The effect of DFuc-90 on intracellular reactive oxygen species (ROS) in bacteria was determined by using ROS detection kit [36]. The polysaccharides were mixed into bacterial suspension with the final concentrations of Fuc, chitosan system was 2 × MIC and 1 × MIC, respectively. The DFuc-90 systems were 2 × MIC and 1 × MIC. LB medium was used as the blank control. Reaction solutions were added to fluorescent probe DCFH-DA to 10 μmol/L and incubated at 37 °C in a shaker (180 rpm) for 1 h. Then, bacteria were collected (4 °C, 8000 rpm, and 5 min) and washed with sterile PBS three times. Bacteria were resuspended in 0.5 mL sterile PBS and the OD_600_ values were measured. Intensity of fluorescence with excitation wavelength at 488 nm and emission wavelength at 525 nm were detected. The effect of DFuc-90 on bacterial intracellular ROS was investigated according to Formula (4).

(4) $$\mathrm{Percentage}\;\mathrm{of}\;\mathrm{bacterial}\;\mathrm{intracellular}\;\mathrm{reactive}\;\mathrm{oxygen}\;\mathrm{species}\;(\%)=\frac{Fluorescence\;values/{OD}_{600}(Fuc/DFuc-90/Chitosan)}{Fluorescence\;values/{OD}_{600}(Control)}\times 100\%$$

#### 3.9.6. Bacterial Intracellular ATP

The effect of DFuc-90 on bacterial intracellular ATP content was evaluated by bac titer-Glo ™ microbial cell viability assay [54]. The bacteria were incubated in the medium with polysaccharide for 5 h at 37 °C. The final concentrations of Fuc, chitosan system were 2 × MIC and 1 × MIC, respectively. The DFuc-90 systems were 2 × MIC and 1 × MIC. LB medium was used as the blank control. After 5 h, 100 μL bacteria suspension and 100 μL kit reagent were mixed into 96-well blackboards, reacting for 5 min. Their OD_600_ values were measured, and the luminescence values were recorded. The percentage of ATP levels was calculated with the following Formula (5).

(5) $$\mathrm{Percentage}\;\mathrm{of}\;\mathrm{bacterial}\;\mathrm{intracellular}\;\mathrm{ATP}\;(\%)=\frac{Fluorescence\;values/{OD}_{600}(Fuc/DFuc-90/Chitosan)}{Fluorescence\;values/{OD}_{600}(Control)}\times 100\%$$

### 3.10. Statistical Analysis

All measurements were performed three times, and the data were expressed as mean ± standard deviation (SD). All statistical analyses were performed using SPSS software 26.0 (SPSS Inc., Chicago, IL, USA). One-way analysis of variance (ANOVA) followed by Tukey’s test was used to determine statistical comparisons between the mean values s, *p* < 0.05 was considered significant.

## 4. Conclusions

In this study, the UV/H_2_O_2_ system was used to degrade fucoidan, decrease its molecular weight, and enhance its antibacterial activity, without changing the main functional group types of fucoidan. Fucoidans before and after degradation were typical sulfated polysaccharides and were mainly composed of fucose, galactose, and some glucuronic acid. DFuc-90 (degraded after 90 min) showed the strongest antibacterial activity against *E. coli* and *S. aureus*. The MIC of DFuc-90 against *E. coli* and *S. aureus* were 4 mg/mL and 8 mg/mL, respectively. It could destroy the bacterial cell wall, lead to intracellular oxidative stress, and reduce ATP synthesis. The higher the polysaccharide concentration, the better the antibacterial activity. Therefore, UV/H_2_O_2_ degradation could be explored as an effective way to degrade polysaccharides and improve their antibacterial activity.

## Figures and Tables

**Table 1:** The average molecular weights of the degraded fucoidans.

Sample	Average Molecular Weights (kDa)
Fuc	530.7
DFuc-15	121.8
DFuc-30	88.3
DFuc-45	47.7
DFuc-60	39.6
DFuc-75	26.7
DFuc-90	23.2
DFuc-120	3.6
DFuc-150	3.5
DFuc-180	3.4

**Table 2:** The chemical compositions of degraded fucoidans.

Sample	Total Sugar (%)	Uronic Acid (%)	Protein (%)	Sulfate Group (%)
Fuc	73.9 ± 2.0 ^a^	9.1± 0.8 ^a^	6.4± 0.2 ^d^	26.9 ± 0.2 ^d^
DFuc-15	72.4 ± 1.3 ^a^	7.9 ± 0.6 ^b^	8.3± 0.5 ^bc^	26.6 ± 0.8 ^d^
DFuc-30	62.1 ± 2.2 ^b^	7.7 ± 0.7 ^b^	8.0 ± 0.5 ^abc^	27.1 ± 1.0 ^cd^
DFuc-45	61.9± 2.6 ^b^	7.6 ± 0.6 ^b^	8.7 ± 0.7 ^a^	27.1 ± 0.9 ^bcd^
DFuc-60	60.4 ± 3.3 ^c^	7.7± 0.5 ^b^	8.5 ± 0.1 ^ab^	27.4 ± 0.5 ^bcd^
DFuc-75	59.6 ± 2.3 ^c^	7.6± 0.7 ^b^	7.5 ± 0.8 ^c^	27.5 ± 0.6 ^bcd^
DFuc-90	61.2 ± 3.8 ^b^	7.5 ± 0.6 ^b^	7.7 ± 0.7 ^bc^	28.4 ± 0.3 ^ab^
DFuc-120	59.6 ± 2.2 ^bc^	7.3± 0.5 ^bc^	7.3 ± 0.6 ^cd^	28.2 ± 0.4 ^abc^
DFuc-150	59.2 ± 3.4 ^c^	5.9± 0.5 ^d^	7.9 ± 0.9 ^c^	28.5 ± 0.5 ^d^
DFuc-180	56.4± 4.4 ^c^	6.5 ± 0.2 ^c^	8.1 ± 0.5 ^abc^	28.7 ± 0.4 ^d^

Different lowercase letters in the same column mean significantly different (*p* < 0.05). Data are presented as the mean ± standard deviation (SD) of three independent experiments.

**Table 3:** The molar percentage of monosaccharides of degraded fucoidans.

Sample	Fucose	Rhamnose	Arabinose	Galactose	Glucose	Mannose	Xylose	Galacturonic Acid	Glucuronic Acid
Fuc	39.1	1.7	1.0	44.4	1.4	2.9	1.1	0.0	8.5
DFuc-30	40.3	1.2	0.9	45.5	1.3	2.7	1.0	0.0	7.1
DFuc-90	41.0	0.8	1.0	43.4	1.9	3.3	0.8	0.3	7.5
DFuc-180	44.9	1.7	1.0	39.5	2.3	4.7	0.0	0.8	5.1

## Data Availability

Data are available in a publicly accessible repository.

## Supplementary Materials

The following supporting information can be downloaded at: https://www.mdpi.com/article/10.3390/md22050209/s1, Figure S1: The diameters of the antibacterial zones of DFuc-90 against *S. aureus* (a) and *E. coli* (b).
