# Supplementary material for: Effects of UV/H2O2 Degradation on the Physicochemical and Antibacterial Properties of Fucoidan
Source: Mar Drugs. 2024 May 3;22(5):209. doi: 10.3390/md22050209 (PMC11123097; doi:10.3390/md22050209)
Supplement: Supplementary file 1 [file marinedrugs-22-00209-s001.zip › marinedrugs-2957303-supplementary.pdf]

(A)

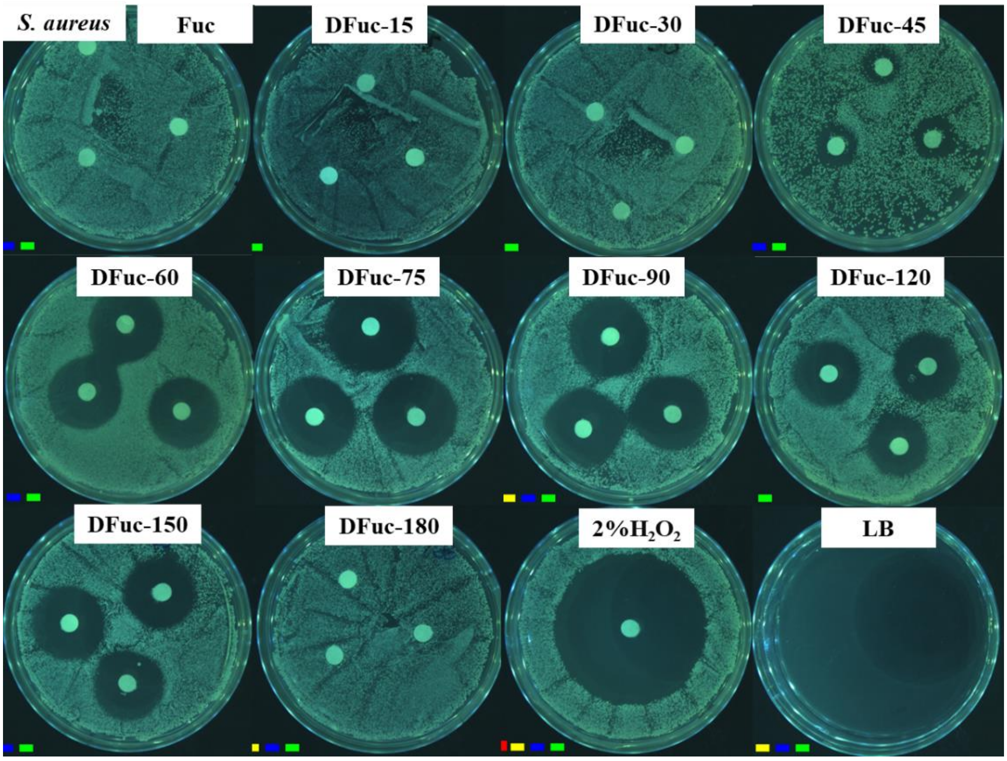


(B)

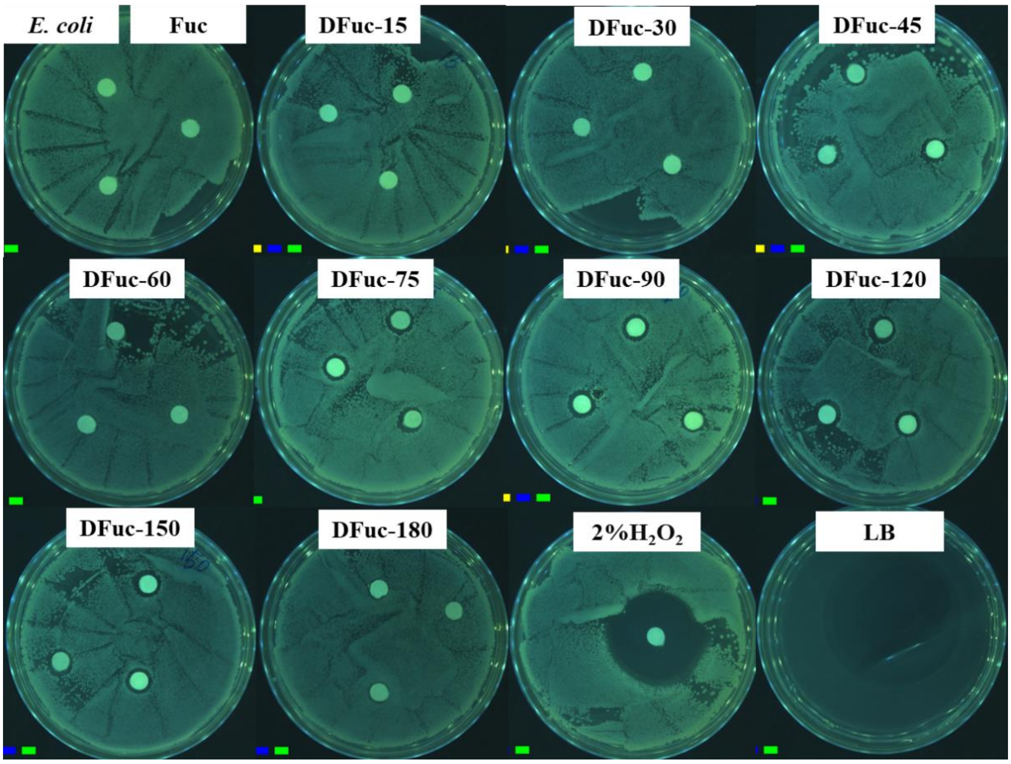


**Figure S1.** The diameters of the antibacterial zones of DFuc-90 (20 mg/mL) against *S. aureus* and *E. coli*. (A) *S. aureus*; (B) *E. coli*.
